# cGMP becomes a drug target

**DOI:** 10.1007/s00210-012-0730-6

**Published:** 2012-02-03

**Authors:** Jens Schlossmann, Elisabeth Schinner

**Affiliations:** Lehrstuhl für Pharmakologie und Toxikologie, Institut für Pharmazie, Universität Regensburg, Universitätsstr. 31, 93040 Regensburg, Germany

**Keywords:** cGMP, Guanylyl cyclases, Guanylate cyclases, cGMP-dependent protein kinase, sGC stimulator, sGC activator, Natriuretic peptides, Pulmonary hypertension

## Abstract

Cyclic guanosine 3′,5′-monophosphate (cGMP) serves as a second messenger molecule, which regulates pleiotropic cellular functions in health and disease. cGMP is generated by particulate or soluble guanylyl cyclases upon stimulation with natriuretic peptides or nitric oxide, respectively. Furthermore, the cGMP concentration is modulated by cGMP-degrading phosphodiesterases. Several targets of cGMP are utilized to effect its various cellular functions. These effector molecules comprise cGMP-dependent protein kinases, ion channels, and phosphodiesterases. During the last decade, it emerged that cGMP is a novel drug target for the treatment of pulmonary and cardiovascular disorders. In this respect, several drugs were developed, which are now in clinical phase studies for, e.g., pulmonary hypertension or cardiovascular diseases. These new drugs act NO-independently with/without heme on soluble guanylyl cyclases or induce subtypes of particular guanylyl cyclases and thereby lead to new therapeutic concepts and horizons. In this regard, the fifth cGMP meeting held in June 2011 in Halle, Germany, comprised the new therapeutic challenges with the novel functional and structural concepts of cGMP generating and effector molecules. This report summarizes the new data on molecular mechanisms, (patho)physiological relevance, and therapeutic potentials of the cGMP signaling system that were presented at this meeting.

The second messenger cyclic GMP (cGMP) is known over 50 years, but its general recognition was overshadowed for a long time by the second messengers cyclic AMP and Ca^2+^. Interest in cGMP increased when it was discovered that NO activates soluble guanylyl cyclase and raises thereby intracellular cGMP concentrations. Soon thereafter, it was found that organic nitrates, compounds that were used to treat angina pectoris, released NO and enhanced cellular cGMP levels. Already in the early 1970, the first target of cGMP was identified, cGMP-dependent protein kinase (cGK), and 15 years later, the second target, the cGMP-gated ion channel in the retina, the first described cyclic nucleotide-gated ion channel (CNG channel). About this time, it was known that cGMP could regulate the cellular concentration of cAMP through several phosphodiesterases (PDEs). Between 1980 and 1985, it was shown that the atria released a peptide, now known as atrial natriuretic peptide (ANP) that caused diuresis and blood pressure lowering by activation of the particulate guanylyl cyclase, a membrane bound enzyme, which is not activated by NO. Since then, it became clear that the cGMP signaling machinery is an important regulatory system in human conditions such as fear processing, cerebellar motor control, pulmonary hypertension, cardiac failure, hypertension, and erectile dysfunction. The rapidly expanding cGMP field had its first successful gathering in 2003 that suggested that new compounds may be available that elevated cellular cGMP levels and could emerge as valuable new therapeutic options in some of the above mentioned diseases. Since then, the cGMP field developed significantly by the analysis of various mouse models, crystal structures, and new regulatory pathways modulated by oxygen radicals. In 2011, the fifth cGMP symposium was held in Halle (Saale), Germany (www.cyclicgmp.net). This report summarizes the new data on molecular mechanisms, (patho)physiological relevance, and therapeutic potentials of the cGMP signaling system that were presented at this meeting. We apologize to our colleagues whose contributions were not enclosed because of space limitations.

## Clinical importance of the particulate guanylyl cyclase /cGMP signaling pathway

**Fig. 1:**
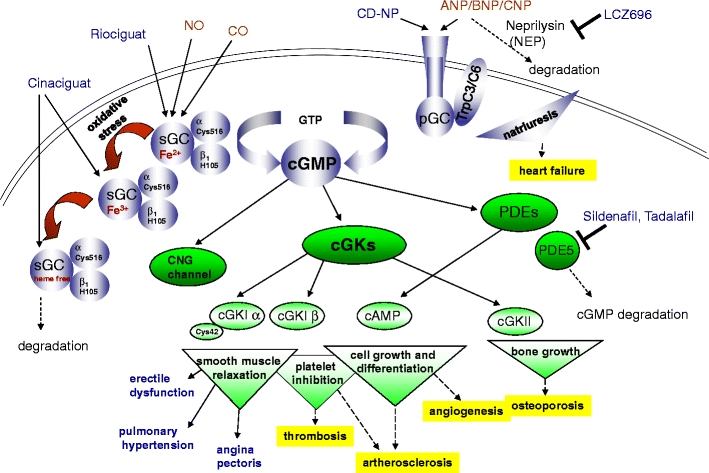
Overview of cGMP signaling from their generation to their effects. An increase in cGMP can be achieved by endogenous cGMP generators (*brown*) or by cGMP-modulating drugs (*blue*). The targets (*green*) of cGMP can regulate downstream pathways and cellular functions (smooth muscle relaxation, platelet inhibition, cell growth and differentiation, bone growth). This suggests potential (*yellow box*) and established indications for cGMP-elevating drugs (*blue*). *ANP/BNP/CNP* atrial/B-type/C-type natriuretic peptide, *CD-NP* cenderitide, *sGC* soluble guanylyl cyclase, *pGC* particulate guanylyl cyclases, *CNG *channel cyclic nucleotide gated ion channel, *PDEs* phosphodiesterases, *cGKs* cGMP-dependent protein kinases

Particulate guanylyl cyclases (pGC) are plasma membrane integrated natriuretic peptide receptors, which contain an extracellular ligand-binding domain, a single transmembrane region, and an intracellular guanylyl cyclase domain (Fig. 1). pGC comprise a protein family of at least seven receptors (GC-A through GC-G) with a diverse natriuretic peptide interaction profile (Nishikimi et al. 2011). GC-A is stimulated by atrial and B-type natriuretic peptide (ANP and BNP), whereas GC-B is activated by C-type natriuretic peptide (CNP), and GC-C is initiated by guanylin and uroguanylin (Kuhn 2009). Hence, the different natriuretic peptides exert diverse physiological functions. ANP and BNP regulate cardiovascular homeostasis. In this regard, BNP and its N-terminal fragment NT-proBNP is a sensitive diagnostic and prognostic marker for heart failure and kidney diseases (Taub et al. 2010). CNP is also involved in cardiovascular function (Afzal et al. 2011). Additionally, CNP augments bone growth as revealed in CNP^−/−^ and GC-B^−/−^ mice (Yasoda and Nakao 2010). Using transgenic and knockout mice, Kazuwa Nakao (Kyoto, Japan) has elucidated that CNP is a potent stimulator of endochondral bone growth. In humans, loss-of-function mutations in the gene coding for GC-B have been proved to be the cause of acromesomelic dysplasia. Following these results, he has started to translate the stimulatory effect of CNP on endochondral bone growth into the therapy for patients with skeletal dysplasias. Nakao has shown that targeted overexpression of CNP in cartilage or systemic administration of CNP reverses the impaired skeletal growth of mice model of achondroplasia, the most common form of human skeletal dysplasias.

Nesiritide, a recombinant form of human BNP, is approved for use in the treatment of heart failure in the USA (Boerrigter et al. 2009). However, despite its favorable cardiorenal properties (e.g., natriuresis, diuresis, inhibition of renin and aldosterone, antihypertrophic, antifibrotic effects, etc.), BNP also serves as a potent vasodilator and may decrease renal perfusion and compromise renal function in heart failure patients. Thus, there is a need for novel NPs with favorable cardiorenal properties. John Burnett (Rochester, NY, USA) described a chimeric NP, CD-NP (cenderitide), comprising CNP-22 and the C terminus of Dendroaspis NP (DNP) (McKie et al. 2010) (Fig. 1). CD-NP activates GC-A and GC-B receptors, increases natriuresis, urinary flow, and glomerular filtration rate, yet is less hypotensive than BNP. In patients with acute heart failure, continuous infusion of CD-NP unloaded the heart without altering blood pressure. Therefore, designer NPs offer advantages over natural NPs and may represent a novel therapeutic strategy in the treatment of heart failure.

Natriuretic peptides are degraded by neprilysin (NEP) (Fig. 1). Hence, increasing the concentration of natriuretic peptides through neprilysin inhibition represents a potential therapeutic approach for cardiac, vascular, and renal protection (Ruilope et al. 2010). However, the clinical benefits can be increased by simultaneous inhibition of NEP and the renin–angiotensin–aldosterone system. LCZ696 is a first-in-class angiotensin receptor neprilysin inhibitor, which inhibits concomitantly NEP and the angiotensin receptor. Martin Lefkowitz (East Hanover, USA) presented the blood pressure lowering effects of LCZ696 in a large phase II study in hypertensive patients (Ruilope et al. 2010). In chronic heart failure, patients treated with LCZ696 increased plasma cGMP levels while decreasing plasma NT-proBNP and plasma aldosterone levels. An ongoing phase III study (PARADIGM-HF) evaluates whether LCZ696 is superior to enalapril in delaying the time to cardiovascular mortality or hospitalization in patients with heart failure. Dual inhibition of neprilysin and the angiotensin receptor with LCZ696 may represent an attractive therapeutic approach for a range of cardiovascular diseases, including hypertension and heart failure.

Hypertension is still a major cause for stroke, heart failure, myocardial infarction, and chronic kidney disease. Blood pressure setting includes hereditary features, but genetic factors have been difficult to identify at the population. Human genetic studies could indicate novel physiological mechanisms that underlie blood pressure. Christopher Newton-Cheh (Boston, MA, USA) used a candidate gene association study of common variants across the NPPA-NPPB locus to identify genetic variants that influence atrial natriuretic peptide (ANP) and B-type natriuretic peptide (BNP) level (Kato et al. 2011; Newton-Cheh et al. 2009a,b). The minor alleles of three noncoding SNPs were associated with higher amounts of ANP and BNP in 14,515 individuals of European ancestry. Two alleles correlating with higher ANP/BNP concentrations were associated with lower systolic and diastolic blood pressure as well as lower odds of hypertension in 29,717 individuals. Thus, the ANP-BNP/pGC/cGMP axis is important in the regulation of BP in humans (Ehret et al. 2011). Further ongoing genome wide association studies (GWAS) have identified additional novel gene loci. These findings provide new insights into the genetics and biology of blood pressure regulation and suggest potential novel therapeutic pathways for cardiovascular disease prevention.

## Clinical importance of the soluble guanylyl cyclase/cGMP signaling pathway

Soluble guanylyl cyclases (sGC) are intracellularly located heterodimers consisting of an α-subunit (α1 or α2) and a heme-containing β-subunit (β1 or β2). The α1β1 and the heterodimer is the dominantly found sGC in most tissues, whereas α2β1 is the major sGC in brain, uterus, and placenta. NO activates sGC by binding to the heme-moiety and thereby inducing cGMP synthesis. The search of new drugs that enhance sGC activity lead to two different classes of compounds: sGC stimulators, which act NO independently and heme dependently, and sGC activators, which function NO and heme independently.

Riociguat is the first drug of the sGC stimulators (Stasch et al. 2011) (Fig. 1). Riociguat has a dual mode of action: It sensitizes sGC to the body’s own NO and can also increase sGC activity in the absence of NO, causing vasorelaxation, antiproliferation, and antifibrotic effects. This is thought to be important because the NO levels in the pulmonary circulation are decreased in patients with pulmonary hypertension (PH). With its novel mode of action, riociguat improves cardiac and pulmonary hemodynamics and has the potential to overcome the limitations of currently approved pulmonary arterial hypertension (PAH) therapies and holds promise in other forms of PH, where no treatment is approved including chronic thromboembolic pulmonary hypertension (CTEPH), PH within left ventricular disease, and PH within interstitial lung disease (PH-ILD). Riociguat is an orally active sGC stimulator that has been shown to have a favorable tolerability profile. Jürgen Behr (Bochum, Germany) presented the results of riociguat in phase II clinical trial in PAH and CTEPH (Ghofrani et al. 2010) as well as in PH within chronic obstructive pulmonary disease (PH-COPD) (Ghofrani et al. 2011) and PH-ILD (Hoeper et al. 2010). The results of these studies suggest that riociguat is a potential treatment option for all of these diseases. As a result, phase III clinical trials have been launched for PAH and CTEPH.

Marco Guazzi (Milano, Italy) further confirmed the importance of elevating myocardial cGMP in the treatment of diastolic heart failure demonstrating an ability of the PDE5 inhibitor, sildenafil, to show reduction in pulmonary vascular tone and RV hemodynamic burden. Significantly improved pulmonary hemodynamics, right ventricular contractility, and chamber dimensions were observed at 6 and 12 months of follow-up, which was paralleled by an improvement in quality of life and clinical status (Guazzi et al. 2011). Further large-scale morbidity and mortality studies are needed to approve whether PDE5 inhibition may effectively impact the appearance of heart failure due to diastolic origin.

Intravascular red cell hemolysis affects NO-redox homeostasis and causes endothelial dysfunction, platelet activation and vasculopathy. The released cell-free plasma hemoglobin reacts with NO in a 1:1 ratio (Donadee et al. 2011). This NO consumption alters vascular function in subjects with sickle cell anemia. Marc Gladwin (Pittsburg, PA, USA) investigated the therapeutic potential to bypass NO scavenging via direct pharmacological induction of sGC. He showed that sGC activators or sGC stimulators could reverse free-hemoglobin-mediated vasoconstriction. The results propose new mechanisms for endothelial injury and impaired vascular function associated with red blood cell storage lesions and hemolysis. Furthermore, theses studies might indicate a new therapeutic approach restoring cGMP levels during conditions, which excite NO scavenging.

Oleg Evgenov (Boston, MA, USA) suggested that stimulation of sGC might represent a new modality for treating pulmonary fibrosis and related conditions as patients with pulmonary fibrosis develop pulmonary hypertension (PH), in part due to impaired production of endogenous NO that activates sGC. Pharmacological stimulation of sGC with riociguat attenuates pulmonary fibrosis, PH, right ventricular hypertrophy, and mortality in bleomycin-exposed mice (Evgenov et al. 2011).

An extraordinary Evening Lecture has been presented by Paul Vanhoutte (Hongkong, China) on endothelial dysfunction and vascular disease. Endothelial cells regulate vascular tone by releasing various contracting and relaxing factors including NO, arachidonic acid metabolites (derived from cyclooxygenases, lipoxygenases, and cytochrome P450 monooxygenases), reactive oxygen species, and vasoactive peptides. Additionally, another pathway associated with the hyperpolarization of the underlying smooth muscle cells plays a predominant role in resistance arteries. Endothelial dysfunction is a multifaceted disorder, which has been associated with hypertension of diverse etiologies involving not only alterations of the NO/sGC/cGMP pathway but also reduced endothelium-dependent hyperpolarizations and enhanced production of contracting factors, particularly vasoconstrictor prostanoids. Vanhoutte (2009) highlighted these different endothelial pathways as potential drug targets for novel treatments in hypertension and the associated endothelial dysfunction and end-organ damage.

## Structures of key members of the GC/cGMP signaling pathway

New insights were elucidated in the protein structures and regulatory pathways of the GC/cGMP system, which enhance the understanding of the (patho)physiological consequences of this signaling pathway. Mammalian soluble guanylyl cyclase (sGC) is a heterodimer consisting of an α- and β-subunit (see above). The C terminus of each subunit includes a catalytic domain, and the active site is arranged from both subunits. The subunits contain a PAS-like domain and a predicted helical region^,^ (Derbyshire and Marletta 2009; Mergia et al. 2009). N-termini of α- and β-subunits are homologous to the H-NOX (heme-nitric oxide/oxygen) family of proteins. The N terminus of the β-subunit contains a ferrous heme cofactor acting as a NO receptor. Ferric heme-oxidized sGC has low activity. The NO complex of the re-reduced heme generates a desensitized, low-activity state of sGC. The molecular mechanism for this desensitization involves site specific S-nitrosation. sGC activation induce slight and complex conformational changes, which are still only partially understood. Some insights were revealed by analyzing the complexes of cinaciguat (BAY 58-2667), a sGC activator, bound to Nostoc H-NOX domain, a homolog of sGC (Martin et al. 2010). Heme forms a covalent inhibitory bond between the Fe and H105 that is part of the αF helix. Cinaciguat does not form this covalent bond yet mimics the rest of the heme features and activates the heme free sGC. Cinaciguat is in this respect similar to protoporphyrin IX that lacks Fe, but is also a good sGC activator. These insights are crucial for the understanding of the action of the new drugs. Hence, further detailed structural and mechanistic analysis will be an important field to enhance the knowledge of sGC physiology.

Much more information is available for protein kinases, which are one of the targets of cGMP. As these kinases are so important not only for physiology but also for disease phenotypes, many kinase structures have now been solved. Our understanding of the structure–function relationship of these enzymes is based on the two seminal papers by Susan Taylor on the structure of cAMP kinase (Knighton et al. 1991; Su et al. 1995). Mammalian protein kinases are highly regulated and are molecular switches that can be turned on and off by various signals. Each kinase consists of two structurally and functionally distinct lobes, the N- and the C-lobe (Taylor and Kornev 2011). The N-lobe contains the ATP binding site and moves upon activation to the C-lobe that harbors the catalytic machinery that transfers the γ-phosphate from the ATP to the substrate peptide. The structures are conserved in all mammalian kinases. They contain an activation loop that is regulated by phosphorylation in ways that are unique for each kinase. Early results on the assembly of cAMP kinase, a tetramer of two regulatory and two catalytic subunits, were discussed. We are at the beginning to understand the supramolecular complexes of cAMP kinase, A kinase anchoring proteins (AKAPs), and their specific localization.

This vivid overview on the structure of protein kinases were an excellent introduction to the following talks on partial structures of cGKI. cGKI is widely expressed in mammalian tissues and modulates a number of biological readouts including vasodilation, motility, and memory (Hofmann et al. 2009). cGKI is a homodimer. Large conformational changes are induced by the cooperative binding of cGMP (Alverdi et al. 2008; Zhao et al. 1997). cGKI is preferentially activated by cGMP at approximately 100-fold lower concentrations than cAMP. cGK can be specifically stimulated in cells and tissues upon exogenous application of lipophilic membrane permeable cGMP analogues (Werner et al. 2011). So far, only the structure of the amino-terminal dimerization domain (aa 1–55 of cGKIβ) has been solved (Casteel et al. 2010). This study confirmed the leucine zipper structure of the dimerization site (Landgraf et al. 1990). Kim and colleagues investigated the interaction between a crystal of the cGMP binding site A of cGKIβ with cAMP and cGMP. Both nucleotides bind to the structure with a two-fold preference for cGMP. Interestingly, cGMP binds in the *syn*, whereas cAMP binds in the *syn* or *anti* configuration. Other parts of the kinase are needed to yield cyclic nucleotide specificity. This problem was investigated by Dostmann’s group that reported the crystal structure of a regulatory domain fragment (aa 78–355 of cGKIα). The fragment encloses the tandem cGMP binding sites (Osborne et al. 2011). The structure contains two separated cGMP binding sites connected by a central helix. The structure revealed a previously unknown helical domain, named switch helix that promotes the assembly of two cGKIα78-355 protomers. Evidence was presented that the switch helix is the critical structure for communication between both subunits. Furthermore, it was suggested that the cGMP binding sites of protomer A regulate the catalytic domain of protomer B.

## Signaling in the cardiovascular system through cGMP and cGKI

Up to the cGMP 2009 meeting in Regensburg (Germany), an unresolved question was whether or not a heme-free sGC exists in vivo. This enzyme cannot be activated in vivo by NO but should respond to the sGC activator cinaciguat. Generation of a heme-free sGC would allow to differentiate physiological functions of NO that are mediated by sGC and that are mediated by radicals (ROS). Peter Brouckarts group in Ghent generated a mouse in which histidine 105 of the β_1_sGC subunit was mutated to a phenylalanine (apo-sGC). The sGC of this mouse line does not respond to NO but does still respond to cinaciguat demonstrating that a heme-free sGC can exist in vivo. This is an excellent proof for the pharmacological significance of sGC activators in humans. NO-dependent effects that need an intact sGC are blood pressure regulation and inhibition of platelet aggregation. In addition, basal sGC activity is essential for life because mice carrying the apo-sGC mutation die premature (*t* ^1/2^ = 30 weeks). sGC is required for relaxation of vascular and stomach smooth muscle but not for relaxation of colon smooth muscle. Further complicating this field was the observation that mice with an intestinal smooth muscle specific deletion of the β_1_sGC show “normal” NO/8 Br-cGMP-dependent relaxation (Groneberg et al. 2011). Even more surprising was the report that β_1_sGC-KO mice have no NO/8 Br-cGMP-dependent relaxation of the corpus cavernosum but are fertile at a normal level confirming a previous finding with cGKI-KO mice (Hedlund et al. 2000).

The focus shifted to the important question: Are cGMP and cGKI protective in cardiac ischemia and hypertrophy? Previous work suggested that cardiomyocyte cGMP and cGKI are essential (Burley et al. 2007; Takimoto et al. 2005). Justin Bice (Cardiff) showed that stimulation of cardiac sGC limits infarct size. More differentiated experiments using cardiomyocyte-specific deletion of cGKI and a postconditioning protocol after ischemia revealed that only treatment with cinaciguat required cGKI, whereas treatment with an A_2B_ receptor agonist and the mitochondria-targeted S-nitrothiol (MitoSNO) were not affected by the absence of cGKI. These findings suggest that the beneficial effects of ischemic postconditioning, activation of the A_2B_ receptor and the direct NO effects via mitochontrial S-nitrosylation are independent of cardiomyocyte cGKI (Krieg, Cambridge). However, cGKI is required to reduce kidney fibrosis induced by ureter ligation (Schlossmann, Regensburg). Eiki Takimoto (Baltimore, MD, USA) reexamined by which mechanism enhanced cGMP-cGKI signaling by PDE5 inhibitor sildenafil ameliorates cardiac maladaptive hypertrophy/remodeling (Takimoto et al. 2005). He concluded from extensive studies that enhanced cGMP-cGKI signaling by sildenafil improves cardiac energetic by restoring mitochondrial respiration a finding that fits to the hypothesis that cGKI (but see results of Krieg) improves mitochondrial respiration by an unknown mechanism. Enrico Patrucco, (München, Germany) showed very convincing results that sildenafil may affect myofibroblast but not cardiomyocyte functions, a result that is in line with the finding that PDE5 is not present in cardiomyocytes, but in cardiofibroblasts (Lukowski et al. 2010).

These results were extended by Philip Eaton (London, UK) and Beate Spießberger (München, Germany). Oxidation of cysteine 42 of GKIα activates the kinase (Burgoyne et al. 2007). A mouse line, in which Cys42 is mutated to a serine, is hypertensive (Prysyazhna et al. 2012). Vascular cGKIα is not activated by H_2_O_2_ in these mice, indicating that eventually redox regulation of the kinase is a physiological event and prevents hypertension. Even more surprising was the report that neuronal cGKI is required to allow proton induced bicarbonate secretion in the duodenum. Neuronal cGKI-KO mice develop duodenal ulcera because the acid chyme of the stomach is not neutralized (Singh et al. 2012). Michael Mendelsohn, now at Merck & Co, gave an intriguing and lively talk on the importance of cGKI for cardiovascular physiology. He pointed out that cGKI have several targets including ion channels, G protein coupled receptors, SR proteins, protein phosphatase subunits, regulators of G proteins, and small G proteins. The variety of these targets allows a variable regulation of cellular and organ functions as needed by the organism. He also showed that Merck is developing sGC stimulators and sGC activators to be used for the treatment of pulmonary hypertension, heart failure, and other diseases. The competition for the best compounds and treatment is now open (Stasch et al. 2011).

## Other functions of cGMP

NO/cGMP has diverse functions in various systems including the cardio-vasculature, neurotransmission, inflammation, and cell death. This part of the meeting covered some of the emerging roles of this pathway.

Sildenafil is an inhibitor of the cGMP-specific phosphodiesterase type 5 (PDE5), which was launched by Pfizer in 1998. The first indication was erectile dysfunction. Later, it was also approved for treating pulmonary hypertension. Interestingly, sildenafil may also have cardioprotective effects in the mdx mouse. The mdx mouse lacks dystrophin and develops cardiac dysfunction as humans with Duchene muscular dystrophy (DMD). DMD is caused by lack of dystrophin (Finsterer and Stollberger 2003) that prevents nNOS expression and signaling. It was therefore likely that increasing cGMP levels might alleviate the symptoms of DMD (Adamo et al. 2010). Joseph Beavo (Washington, DC, USA) reported that, in contrast to sildenafil, the specific PDE5 inhibitor tadalafil does not mediate cardioprotection in mdx mice. He pointed out that PDE5 is not expressed in adult mouse cardiomyocytes, where, however, PDE1C is highly expressed. While sildenafil at high concentrations inhibits PDE1C, tadalafil does not. Thus, Beavo suggested that, in cardiomyocytes, PDE1C is the target for sildenafil, which may be applied as a preventive and curative treatment for DMD-caused cardiomyopathy.

In the nervous system, NO acts as a retrograde messenger that is generated postsynaptically to increase the neurotransmitter release presynaptically. This role of NO is thought to play a role in the long-term potentiation (LTP) and modulation of synaptic transmission (Haghikia et al. 2007). The group of Doris Koesling (Bochum, Germany) investigated the role of sGC in this process. Both α1 and α2 isoforms of sGC are expressed in glutamatergic and GABA-ergic neurons, and deletion of both α-isoforms in mice abolished LTP in the hippocampus and visual cortex. Interestingly, presynaptic glutamate release was only reduced in sGC α1 KO mice, whereas a reduction of postsynaptic NMDA receptor currents was only observed in sGC α2 KO mice. Koesling further suggested that eNOS is the source of NO mediating presynaptic glutamate release, which interacts with signaling through an α1-dependent sGC via cGMP to open hyperpolarisation-activated cyclic nucleotide-gated channels (Neitz et al. 2011).

Another emerging gaseous transmitter, besides NO and CO, with close relations to cGMP is hydrogen sulfide (H_2_S). Andreas Papapetropoulos (Patras, Greece) reported on the regulation of vascular growth and tone by H_2_S. In blood vessels, H_2_S is endogenously synthesized from l-cysteine by the two enzymes cystathionine-synthase (CBS) and cystathionine γ-lyase (CSE). CBS is highly expressed in the central nervous system, while CSE is abundantly present in the heart, lung, blood vessels, liver, and kidney. Cardiovascular functions of H_2_S include antiapoptotic effects on cardiomyocytes, cardioprotective actions, and altered vascular tone (Suzuki et al. 2011). Exogenously administered Na_2_S stimulates endothelial proliferation, migration, and capillary-like network formation. Vascular endothelial growth factor (VEGF) increased H_2_S production in endothelial cells, and genetic or pharmacological CSE inhibition reduced VEGF-driven angiogenic responses (migration, sprouting). Thus, H_2_S could be a target for the treatment of diseases with altered angiogenesis. Recently, it was shown that H_2_S inhibits phosphodiesterase activity in vitro. Furthermore, H_2_S increases cGMP levels in smooth muscle cells (Bucci et al. 2010) and probably modulates vascular tone via cGKI. Thus, H_2_S may play an important role in vascular biology. The functional interactions between NO, cGMP, and H_2_S were extended to the chemical level by Akaike (Kumamoto, Japan). About 80% of H_2_S is present in its anionic form, SH^−^, which he claimed to be a negative regulator of endogenously formed 8-nitro-cGMP. This nitrated cGMP derivative reacts with protein sulfhydryls resulting in adduction of cGMP to proteins, which is a posttranslational modification called protein S-guanylation (Ahmed et al. 2011). 8-Nitro-cGMP is formed from guanine derivatives by reactive nitrogen species stemming from the reaction of NO with ROS (Ahmed et al. 2011). In cells, SH^−^ reacts with 8-nitro-cGMP generating a new electrophilic derivative. This derivative modulates electrophilic signaling via the oncogene H-Ras. H-Ras activation then suppressed cellular senescence. Together, these results suggest novel roles of H_2_S, i.e., interaction of its anionic form with 8-nitro-cGMP. Via this mechanism, H_2_S may also attenuate cardiac dysfunction.

Renate Pilz (San Diego) switched the topic to bone mass and strength. In osteoblasts, fluid shear stress is a major mechanism by which mechanical forces stimulate osteoblast/osteocyte growth and differentiation (Ehrlich and Lanyon 2002). Fluid shear stress activates cGK via NO/cGMP signaling. This results in induction of *fos* family genes mediated through activation of Erk1/2 (Rangaswami et al. 2009). She reported that cGKII, but not cGKI, activates Src in mechanically stimulated osteoblasts, which initiates a proliferative response. This process requires interaction of Src with the mechanosensors of bones, the β3 integrins. It further depends on Src activation, i.e., de-phosphorylation by Src homology 2 domain-containing tyrosine phosphatases (SHP) 1 and 2. SHP-1 is a novel substrate that is directly phosphorylated and activated by cGKII. Furthermore, fluid sheer stress triggers the formation of a novel “mechanosome” composed of cGKII, Src, SHP 1 and 2, and β3 integrins. This newly discovered mechanism of Src activation mediates Erk1/2 activation and finally bone growth. This suggests a novel indication for cGK-activating drugs, i.e., osteoporosis in which they may mimic the anabolic effects of mechanical bone stimulation (Rangaswami et al. 2010).

## cGMP and ion channels

Endothelial NO regulates vascular tone by signaling through sGC, cGMP, and cGK. The most important targets of cGKI include the myosin-interacting subunit of myosin phosphatase 1, the regulator of G-protein signaling 2, the inositol receptor associated cGKI-substrate (IRAG), and the BK channel. Recent evidence suggest that TrpC channels are also targets of cGKI in smooth muscle and mediate, at least partially, the relaxant effects of cGMP (Chen et al. 2009; Kwan et al. 2004; Yuasa et al. 2011). This new concept was tested by investigating the role of cGMP/cGKI signaling on vascular tone and peripheral resistance using cGKI-, TrpC6-, and TrpC3 knockout mice (Wegener, München). However, neither differences were found in the response to alpha-adrenergic stimulation with respect to the contractility of thoracic aorta nor to the increase in peripheral resistance. Activation of cGKI by 8-Br-cGMP diminished aortic tone and peripheral resistance to a similar extent in control, TrpC6^−/−^, and TrpC3^−/−^ mice. No effect of 8-Br-cGMP was observed in preparations from smooth-muscle specific cGKI^−/−^ mice. These results therefore suggest that cGMP/cGKI signaling in aorta and peripheral vessels from mice does not require TrpC6 or TrpC3 channels.

Consistent with this, Michaela Kuhn (Würzburg, Germany) reported on a novel cGMP-independent signaling pathway of pGC-A, the receptor for atrial natriuretic peptide (ANP), involving TRPC3/C6 channels (Klaiber et al. 2011). ANP regulates arterial blood pressure, moderates cardiomyocyte growth, and stimulates angiogenesis and metabolism. ANP has been invoked to trigger a cGMP-dependent signaling pathway that prevents pathological increases in [Ca^2+^]_i_ in cardiomyocytes. In chronic cardiac hypertrophy, ANP levels are markedly increased, and GC-A/cGMP responses to ANP are blunted due to receptor desensitization. Michaela Kuhn showed that in this situation ANP binding to pGC-A stimulates a novel cGMP-independent signaling pathway in cardiomyocytes resulting in pathologically elevated [Ca^2+^]_i_. In this condition, pGC-A forms a stable complex with TRPC3/C6 channels resulting in TRPC3/C6-mediated Ca^2+^ entry followed by stimulation of calmodulin kinase II (CaMKII) to phosphorylate L-type Ca^2+^ channels leading to increased L-type Ca^2+^ channel mediated Ca^2+^ current and a rise in intracellular Ca^2+^ levels (Klaiber et al. 2011). Although these observations are very intriguing, they need confirmation by other groups because they would suggest that treatment of heart failure with ANP/BNP or cGMP elevating drugs may not result in alleviation of the disturbed cardiac situation. Furthermore, under physiological conditions, activation of a cGMP-dependent pathway moderates the Ca_i_^2+^-enhancing action of hypertrophic factors such as angiotensin II (Takimoto et al. 2005).

## Upcoming topics

Heme oxygenase-1 (HO-1) metabolizes heme to equimolar amounts of carbon monoxide (CO), biliverdin and ferrous iron. CO activates sGC and thereby indirectly leads to suppression of neointima formation after arterial injury. Consequently, HO-1 deletion exacerbates lesion development. William Durante and co-workers (Columbia, SC, USA) found that induction of HO-1 or CO application leads to increased cGMP levels and antiproliferative effects in cultured vascular smooth muscle cells (VSMCs). The sGC stimulator YC-1 did not only increase the antiproliferative effect of CO in VSMCs by sGC stimulation but also by induction of HO-1 gene expression and hence increased CO production. This effect could also be reflected in the suppression of neointima formation and increased cGMP levels after vessel injury in vivo. However, the sGC stimulator BAY41-2272 did not influence HO-1 gene induction. Vice versa, HO-1 deficiency also leads to a decline in sGC expression resulting in impaired endothelium-dependent vasorelaxation in response to sGC stimulators and activators. In conclusion, HO-1 elicits important vasoprotective actions by stimulating sGC activity via CO and preserving sGC expression levels.

After showing that the development of nitrate tolerance in vivo is in part mediated by sGC desensitization through nitroglycerin-induced S-nitrosylation of cysteine residues (Sayed et al. 2008), Annie Beuve (New Jersey, USA) presented data also suggesting a role for this mechanism in cardiovascular disease development. The authors used angiotensin II (AT II)-induced hypertension as a model for oxidative cardiovascular disease. AT II treatment induced in rats’ resistance to NO donor-mediated relaxation. Simultaneously, S-nitrosation was globally increased in thoracic aortae of those rats and associated with decreased NO-dependent cGMP production. Notably, sGC expression levels were unchanged in AT II-treated compared to untreated animals. These in vivo findings were supported by in vitro data showing that especially S-nitrosation of cysteine residue 516 in the alpha subunit of sGC mediates AT II-induced NO desensitization independent of heme oxidation. The authors therefore propose that additional to decrease NO bioavailability this desensitization of sGC to NO by S-nitrosation of sGC-Cys516 contributes to the decreased vascular reactivity in AT II-induced hypertension.

Alexander Pfeifer (Bonn) shifted the focus from vascular diseases to obesity. The cGMP pathway can be found in both white and brown fat cells where one of the receptors of cGMP, cGKI, might play a major role in calorie burning in brown fat cells. Differentiation of brown fat cells was markedly suppressed in cGKI deficient mice (Haas et al. 2009), whereas others showed that overexpression of cGKI in fat cells leads to enhanced mitochondrial biogenesis and thereby to prevention of obesity. cGKI is expressed in murine white adipocytes and may be involved in the differentiation of white adipocytes.

Roland Seifert (Hannover) took us to basic science and draw attention to cGMP’s abandoned siblings cCMP and cUMP. Their recent findings that bacterial “adenylyl” cyclase toxins also exhibit cytidylyl- and uridylyl cyclase activity (Gottle et al. 2010) renewed the interest in this field. To evade methodological mistakes that were done in early years of cCMP and cUMP research, a highly sensitive quantitative HPLC-MS/MS was used. The cyclic nucleotides were detected in cultured cell and in human urine by this method. Several cAMP and cGMP-degrading phosphodiesterases have cUMP degrading activity (Reinecke et al. 2011), and some biological effects of cCMP and/or cUMP were indicated in platelet aggregation (Desch et al. 2010) and neuronal cell differentiation. Collectively, those findings argue for a role of cCMP and cUMP as (old) new endogenous second messengers (Beste et al. 2012).

## Conclusion

The cGMP symposia, which are organized since 2003 every 2 years, are outstanding international meetings for researchers in the cGMP field in basic and clinical research. The 5th cGMP symposium, which took place in Halle (Saale), Germany, uncovered that the second messenger molecule cGMP is a valuable drug target for the treatment of various pulmonary and cardiovascular diseases (Fig. 1). Several compounds acting on soluble guanylyl cyclases (sGC stimulators and sGC activators) or particulate guanylyl cyclases (e.g., designer natriuretic peptides or neprilysin/AT-receptor inhibitors) were synthesized, which are now coming into the drug pipeline. In this regard, several human trial studies are performed for the treatment of diverse pulmonary diseases, e.g., COPD, CTEPH, or other PH disorders, and for the treatment of diverse forms of heart failure (Table 1). Furthermore, research of the cGMP generation and signaling mechanisms, structural work, genetic and tissue models were presented and are further needed to elucidate new compounds and novel treatment options in the cGMP field.

**Table 1:** Human trial studies described in the cGMP2011 symposium

Drug	Drug target mechanism	Human trial study	Disease	References
CD-NP	Activator of GC-A/GC-B	Phase II	Acute heart failure	McKie et al. 2010
LCZ696	Inhibition of neprilysin/angiotensin receptor	Phase II	Hypertension	Ruilope et al. 2010
		Phase II: PARAMOUNT	Heart failure	
		Phase III: PARADIGM-HF		
Riociguat	sGC stimulator	Phase II:	PAH, CTEPH	Ghofrani et al. 2010, 2011; Hoeper et al. 2010
			PH-COPD	
			PH-ILD	
		Phase III:		
		PATENT-trial	PAH	
		CHEST-trial	CTEPH	
Sildenafil	PDE5 inhibitor	Phase II: 1 year study	Heart failure	Guazzi et al. 2011
		GWAS analysis: ANP-BNP/pGC/cGMP axis	Hypertension	Newton-Cheh et al. 2009a, b; Ehret et al. 2011; Kato et al. 2011
